# Head and neck cancer N-glycome traits are cell line and HPV status–dependent

**DOI:** 10.1007/s00216-022-04376-x

**Published:** 2022-10-27

**Authors:** Mohammad Rasheduzzaman, Abarna V. M. Murugan, Xi Zhang, Tiago Oliveira, Riccardo Dolcetti, Liz Kenny, Newell W. Johnson, Daniel Kolarich, Chamindie Punyadeera

**Affiliations:** 1 Centre for Biomedical Technology, School of Biomedical Sciences, Faculty of Health, Queensland University of Technology, Kelvin Grove, QLD Australia; 2 Saliva and Liquid Biopsy Translational Laboratory, Griffith Institute for Drug Discovery, Griffith University, Nathan, QLD Australia; 3 Institute for Glycomics, Griffith University, Gold Coast Campus, Southport, 4222 QLD Australia; 4 Peter MacCallum Cancer Centre, University of Melbourne, Melbourne, Australia; 5 Sir Peter MacCallum Department of Oncology, The University of Melbourne, Victoria, 3010 Australia; 6 Department of Microbiology and Immunology, The University of Melbourne, Victoria, 3010 Australia; 7 The University of Queensland Diamantina Institute, Brisbane, QLD Australia; 8 Department of Radiation Oncology, Cancer Care Services, Royal Brisbane and Women′s Hospital, Joyce Tweddell Building, Herston, QLD 4029 Australia; 9 Menzies Health Institute Queensland, Griffith University, Gold Coast, QLD Australia; 10 ARC Centre of Excellence for Nanoscale BioPhotonics, Griffith University, Gold Coast, QLD Australia

**Keywords:** Head and neck cancers, N-glycosylation, Glycomics, Sialic acid, Cell line, HPV

## Abstract

Glycosylation is the most common post-translational modification of proteins, and glycosylation changes at cell surfaces are frequently associated with malignant epithelia including head and neck squamous cell carcinoma (HNSCC). In HNSCC, 5-year survival remains poor, averaging around 50% globally: this is partly related to late diagnosis. Specific protein glycosylation signatures on malignant keratinocytes have promise as diagnostic and prognostic biomarkers and as therapeutic targets. Nevertheless, HNSCC-specific glycome is to date largely unknown. Herein, we tested six established HNSCC cell lines to capture the qualitative and semi-quantitative N-glycome using porous graphitized carbon liquid chromatography coupled to electrospray ionisation tandem mass spectrometry. Oligomannose-type N-glycans were the predominant features in all HNSCC cell lines analysed (57.5–70%). The levels of sialylated N-glycans showed considerable cell line-dependent differences ranging from 24 to 35%. Importantly, α2-6 linked sialylated N-glycans were dominant across most HNSCC cell lines except in SCC-9 cells where similar levels of α2-6 and α2-3 sialylated N-glycans were observed. Furthermore, we found that HPV-positive cell lines contained higher levels of phosphorylated oligomannose N-glycans, which hint towards an upregulation of lysosomal pathways. Almost all fucose-type N-glycans carried core-fucose residues with just minor levels (< 4%) of Lewis-type fucosylation identified. We also observed paucimannose-type N-glycans (2–5.5%), though in low levels. Finally, we identified oligomannose N-glycans carrying core-fucose residues and confirmed their structure by tandem mass spectrometry. This first systematic mapping of the N-glycome revealed diverse and specific glycosylation features in HNSCC, paving the way for further studies aimed at assessing their possible diagnostic relevance.

**Supplementary Information:**

The online version contains supplementary material available at 10.1007/s00216-022-04376-x.

## Introduction

Head and neck cancer (HNC) refers to a heterogeneous group of malignant neoplasms, with over half a million new cases diagnosed annually across the globe [1, 2]. Majority of them originate from lining mucosae and are collectively described as head and neck squamous cell carcinoma (HNSCC). These tumours can originate from the hypopharynx, oropharynx, lip, oral cavity, nasopharynx, or larynx with a range of well-established risk factors [3, 4]. Only about 50% of HNSCC patients live beyond 5 years [5].

Human papillomaviruses (HPV-16 and HPV-18; high-risk types) are small, double-stranded, circular DNA viruses that are responsible for a global epidemic of a subset of HNSCC, mainly originating in the lymphoid mucosa of the oropharynx (oropharyngeal cancers, OPC) [6, 7]. Current diagnostic practise requires an expert visual examination and imaging by X-radiography, CT scan and MRI in conjunction with tissue biopsy staging [8, 9]. Less invasive means to identify, stage and monitor treatment response in HNSCC are required to improve patient outcomes [10]. Glycosylation, a common post-translational modification (PTM) of proteins, has frequently been reported to undergo major changes that are associated with malignant transformation of epithelial cells [10]. Understanding the glycosylation features in most widely used HNSCC cell lines is an important prerequisite to investigate how protein glycosylation can provide novel diagnostic and therapeutic opportunities [2].

Though no comprehensive studies of the HNSCC N-glycome have been published to date, glycosylation changes have been reported in several studies. As an example, in saliva, the total sialic acid/total protein ratio as well as the activities of α2-3 and α2-6 sialyltransferases were reported to be significantly higher in patients with metastatic oral cancer [11]. Additionally, in patient sera, tri-antennary and tetra-antennary N-glycans with varying degrees of sialylation and fucosylation have been reported to be a potential diagnostic biomarker for oral squamous cell carcinoma (OSCC) [12]. Multiple fucosyltransferases such as FUT1, FUT2, FUT3 and FUT6 have also been associated with the high abundance of Lewis Y (Le^y^) and sialyl Lewis X (SLe^x^) epitopes, changes which are known to promote EGFR phosphorylation in OSCC cell lines [13, 14]. In OSCC tumour tissues, an overexpression of *MGAT5* (also known as GNT-V) enhanced CEACAM6 N-glycosylation, which in turn promoted EGFR signalling that correlated with poor prognosis [15]. Despite the above mentioned studies, a detailed map of the HNSCC cell N-glycome is still lacking. Furthermore, it is unknown if and how the expression of HPV infection impacts the HNSCC glycocalyx.

We employed a well-established porous graphitized carbon (PGC) glycomics platform [PGC liquid chromatography (LC) electrospray ionisation tandem mass spectrometry (ESI–MS/MS)] to establish the first N-glycan map of the most widely used HNSCC cell lines (SCC-25, CAL-27, SCC-9, FaDu, 2A3 and VU-147 T), of which 2A3 and VU-147 T considered as HPV ( +) cells. In addition, we have also investigated whether HPV infection affect the relative distribution of N-glycans. While the HNSCC cells analysed exhibited general similarities, distinct cell line-specific N-glycosylation traits were identified that make each cell line an individual research resource. Distinct differences were identified in sialic acid linkage distribution. Various levels of phosphorylated oligomannose, oligomannose and sialylated N-glycans were determined. We also confirmed that oligomannose N-glycans can act as a substrate for FUT8, as demonstrated by the presence of several core-fucosylated oligomannose-type N-glycan structures. This systematic N-glycome map of the most widely used HNSCC cell lines provides the first HNSCC-glycome reference dataset that builds the foundation for future glyco-biomarker research in HNSCC.

## Materials and methods

### Chemicals, reagent and equipment

Ethanol, methanol, glacial acetic acid and direct blue 71, polyvinylpyrrolidone (PVP40), ammonium hydroxide solution (≥ 25% (vol/vol) NH_3_ in H_2_O, sodium borohydride (NaBH4), potassium hydroxide (KOH), trifluoroacetic acid (TFA), LC–MS grade, water, fetuin from foetal calf serum, RPMI medium and foetal bovine serum (FBS) were obtained from Sigma-Aldrich. AG50W-X8 cation-exchange resin was purchased from BioRad. ZipTip with C18 resin and Immobilon-PSQ (0.45 µm pore size) PVDF membranes were obtained from Millipore. RIPA buffer and Pierce™ BCA protein assay kit were purchased from Thermo Fisher Scientific. PNGase F was from New England Biolabs. Porous graphitized carbon columns were obtained from New Objectives (length 150 mm × 100 μm I.D., 5 μm particle size). The SpeedVac concentrator was from Thermo Fisher Scientific. Acetonitrile LC–MS grade was purchased from Merck.

### Origin of cell lines and cell culture

All the HNSCC cell lines were STR profiled and authenticated. This gave us the confidence that our cell lines are correctly identified, and not cross-contaminated with other cells. In addition, we have also performed mycoplasma testing using Lonza’s MycoAlert® Mycoplasma Detection Assays and were confirmed to be of mycoplasma free. Six HNSCC cell lines, including two HPV positive, were purchased from ATCC (American Type Culture Collection) or a generous gift (Supplementary Table 1). Cell culture was performed according to ATCC guidelines. Cells were cultured under standard conditions in humidified incubators at 37 °C, 20% O_2_, 5% CO_2._ Briefly, 0.6–0.7 million cells were seeded into T75 mL flasks and incubated with medium consisting of RPMI-1640, 10% foetal bovine serum and penicillin/streptomycin, of which 2A3 cells were incubated with media consisting of hGlucose DMEM-10%FBS + 2 nM glutamine + G418 200 ug/mL. Once cells density reached to 85–90% confluence, we washed the cells twice with ice cold PBS. Cells were lysed and harvested (by scraping) in cold RIPA buffer with freshly added protease inhibitor cocktails. The next step was to vigorously vortex the cells thrice for 30 s followed by ultrasonication (an ultrasonic bath for 10 min). Cell lysates were centrifuged at 14,000 × g for 15 min at 4 °C. Supernatant was collected, and protein concentrations were measured using the BCA protein assay kit as per the manufacturer’s instruction. After protein quantitation, (glyco)proteins were precipitated using ice cold (− 20 °C) acetone. The resultant protein pellet was allowed to air dry at room temperature. The sample was resuspended using 8 M urea by intensive vortexing.

### Sample preparation and data acquisition

50 µg of (glyco)proteins were immobilised onto a 0.45 µm pore size PVDF, washed and stained with direct blue 71. N-glycans were released from glycoproteins using PNGase F as described previously [16, 17, 18]. Released N-glycans were then further reduced and desalted before subjected to PGC-LC-ESI MS/MS glycomics [16, 17, 18, 19] [see Supplementary Material (Supplementary Fig. 1) for experimental details reported in a MIRAGE (Minimum Information Required for A Glycomics Experiment) compliant manner [20, 21, 22, 23]].

### Data analysis and availability

N-glycan structures were determined as previously described [16, 18, 19]. GlyConnect Compozitor (https://glyconnect.expasy.org/compozitor/) was used to visualise the glycan network [24]. The relative intensities and statistical analysis (supplementary materials, pages 7–14) were determined using GraphPad Prism (v8.4.3) and presented with mean + SD. Bar graphs were generated from six replicates (two biological replicates, of which each group had three technical replicates).

The data is made available via GlycoPOST, accession number GPST000279, URL, https://glycopost.glycosmos.org/preview/7702599506310db315e4be PIN CODE: 4365 [25].

## Results

### HNSCC N-glycome features are cell line dependent

**Fig. 1:**
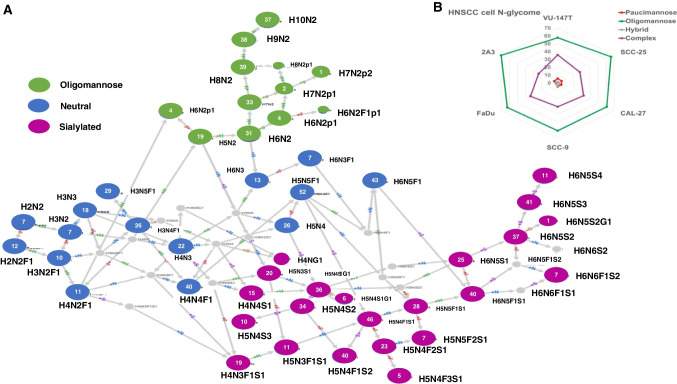
HNSCC N-glycome features are cell line dependent. Quantitative distribution of major glycosyl categories. **A** GlyConnect network in acquired glycans dataset. **B** Quantitative distribution of four major glycan classes in six different HNSCC cells

We profiled the N-glycome from six different, widely used HNSCC cell lines using PGC-nanoLC ESI MS/MS. Overall, we identified 99 different N-glycan structures that were present in 49 compositions (Supplementary Table 2). The integrity of the dataset was first verified using *glyConnect compozitor* network analysis to understand the biosynthetic connections between the identified N-glycans and to uncover any potential gaps in the acquired dataset (Fig. 1A) [24]. These data confirmed the majority of virtual nodes (not present in our dataset, generated by the software) to be derived from compositions unlikely to occur due to currently understood biosynthetic constrains [e.g. Hex4-HexNAc2-dHex1-NeuAc1 (H4N2F1S1)] or from known intermediate structures that are usually quickly processed into other structures (e.g. H4N4, or H5N3, Fig. 1A). This led us to conclude that the acquired dataset was not missing any glycans and that identified N-glycans were in good agreement with our current understanding of the N-glycan biosynthesis.

While the overall compositional profile was similar between the analysed cell lines, we observed a considerable diversity with respect to the quantitative distribution of the individual N-glycan structures (Table 1, Fig. 1B, Supplementary Table 3 and Supplementary Table 9). Oligomannose-type N-glycan levels ranged between 57.5–70% across all analysed cells lines, while complex-type N-glycans made up between 24 and 36% (Fig. 1B, Supplementary Table 5 and Supplementary Table 7). Overall, these two glycan families constituted the major components of the HNSCC cell line N-glycome. The levels of pauci-mannose and hybrid-type N-glycans were low and did in average not exceed 6% of the total N-glycome (Fig. 1B, Table 1 and Supplementary Table 4 and Supplementary Table 6).

**Table 1:** Average relative abundances (derived *n* = 6) of different N-glycan classes for six different HNSCC cell lines. Cell lines labelled with an * are considered HPV + cell lines. Fuc considered as fucose. Standard deviation of data is included

Traits	VU-147 T*	SCC-25	CAL-27	SCC-9	FaDu	2A3*
Paucimannose	5.57(0.43)	4.97(0.66)	4.52(1.53)	4.62(1.42)	1.96(0.20)	4.45(2.11)
Paucimannose Fuc	4.12(0.24)	3.76(0.38)	3.39(0.95)	3.96(1.28)	1.48(0.13)	3.54(1.71)
Oligomannose	57.53(2.19)	66.36(2.26)	60.89(2.38)	61.07(1.07)	62.73(3.06)	70.27(0.66)
Oligomannose Fuc	0.42(0.07)	0.07(0.04)	0.23(0.02)	0.63(0.34)	0.16(0.05)	0.31(0.20)
Oligomannose Pho	5.85(0.85)	1.33(0.51)	3.65(0.31)	5.68(2.10)	2.80(0.68)	4.59(1.10)
Hybrid	1.27(0.18)	1.06(0.04)	2.28(0.61)	3.85(1.17)	1.42(0.11)	1.56(0.38)
Hybrid Fuc	0.32(0.07)	0.27(0.02)	0.86(0.30)	2.21(0.94)	0.42(0.04)	0.67(0.16)
Hybrid Sialyl	0.53(0.10)	0.47(0.04)	1.46(0.37)	2.16(0.62)	1.00(0.09)	1.07(0.21)
Hybrid Neutral	0.74(0.14)	0.59(0.04)	0.82(0.25)	1.68(0.62)	0.41(0.02)	0.50(0.19)
Hybrid α2-6	0.37(0.10)	0.36(0.03)	1.10(0.43)	0.92(0.20)	0.68(0.12)	0.57(0.14)
Hybrid α2-3	0.16(0.02)	0.11(0.02)	0.33(0.08)	1.20(0.48)	0.30(0.05)	0.45(0.07)
Hybrid ND	0.00(0.00)	0.00(0.00)	0.03(0.01)	0.04(0.01)	0.02(0.00)	0.04(0.02)
Hybrid, α2-6 NeuAc, Fuc	0.01(0.01)	0.07(0.01)	0.38(0.22)	0.57(0.28)	0.19(0.01)	0.22(0.04)
Hybrid α2-3 NeuAc, Fuc	0.03(0.01)	0.02(0.01)	0.14(0.05)	0.89(0.42)	0.10(0.04)	0.21(0.03)
Complex	35.63(2.21)	27.61(1.94)	32.31(0.86)	30.46(3.14)	33.90(3.06)	23.71(1.47)
Complex Fucosylated	3.29(0.32)	8.52(1.04)	10.28(0.69)	10.03(2.45)	7.54(1.81)	6.43(1.03)
Complex Sialylated	34.52(2.24)	23.88(2.28)	31.24(0.93)	29.75(3.16)	33.12(3.18)	22.93(1.42)
Complex Neutral	1.11(0.10)	3.73(0.36)	1.07(0.08)	0.71(0.05)	0.77(0.17)	0.78(0.08)
Complex α2-6 NeuAc	5.40(0.55)	8.30(0.33)	9.84(1.91)	6.19(1.71)	9.04(0.46)	5.58(0.74)
Complex α2-3 NeuAc	1.49(0.20)	1.93(0.20)	4.11(1.48)	7.04(2.03)	3.69(1.01)	3.49(0.71)
Complex ND	20.93(1.32)	9.80(1.22)	11.96(0.72)	11.00(2.01)	13.83(2.15)	9.74(0.53)
Complex Mix	6.70(0.45)	3.86(1.20)	5.32(0.44)	5.52(1.43)	6.56(1.75)	4.13(0.29)
Complex, α2-6 NeuAc, Fuc	0.31(0.04)	4.13(0.48)	4.97(1.21)	2.36(0.58)	3.65(0.75)	2.50(0.47)
Complex, α2-3 NeuAc, Fuc	0.70(0.04)	1.08(0.19)	2.74(1.12)	5.76(2.12)	2.17(0.77)	2.51(0.45)
Sialylation total	35.05(2.22)	24.35(2.30)	32.70(1.09)	31.91(2.58)	34.13(3.22)	24.00(1.58)
α2-6 NeuAc total	5.77(0.55)	8.66(0.29)	10.94(2.32)	7.12(1.64)	9.71(0.53)	6.15(0.93)
α2-3 NeuAc total	1.65(0.22)	2.04(0.18)	4.43(1.54)	8.24(2.50)	3.99(1.05)	3.94(0.78)
α2-6 NeuAc + Fuc total	0.33(0.01)	4.20(0.56)	5.35(1.81)	2.93(1.29)	3.84(0.92)	2.72(0.63)
α2-3 NeuAc + Fuc total	0.73(0.01)	1.10(0.24)	2.88(1.37)	6.65(0.05)	2.27(0.98)	2.72(0.54)
Sialylation ND	20.93(1.41)	9.80(1.27)	12.00(0.54)	11.04(0.18)	13.86(2.46)	9.79(0.38)
Mix α2-6,α2-3	6.70(0.47)	3.86(1.54)	5.32(0.18)	5.52(0.42)	6.56(2.17)	4.13(0.25)
Neutral	1.85(0.03)	4.32(0.45)	1.89(0.29)	2.39(0.40)	1.18(0.17)	1.28(0.32)
Fucosylation	8.15(0.32)	12.62(1.15)	14.76(2.42)	16.83(2.02)	9.61(2.41)	10.95(0.96)

### The impact of HPV infection on HNSCC cell glycosylation

**Fig. 2:**
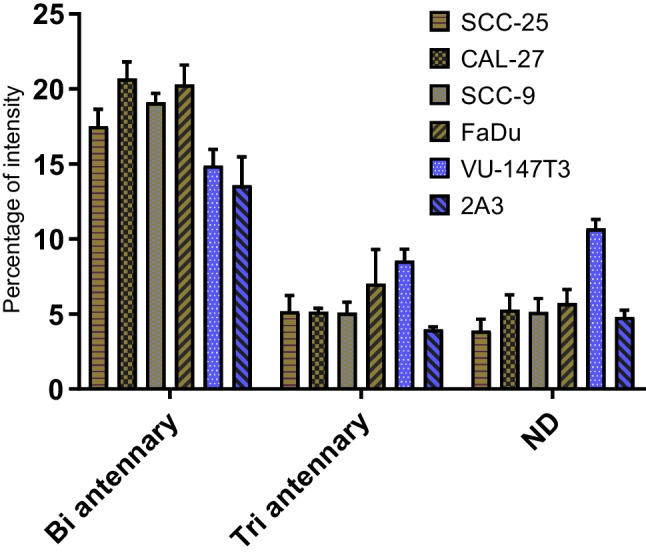
The impact of HPV infection on HNSCC cell glycosylation. Distribution of sialylated glycan types in FaDu and 2A3 cells. Antennary (Bi antennary, Tri antennary) type glycan distribution in different cell lines

Infection with HPV has been associated with an increased risk of developing HNSCC. It is well known that the HPV-positive HNSCC and HPV-negative HNSCC are biologically and clinically different [26, 27]. We then next investigated whether the presence of HPV genome or fragments thereof could impact the overall HNSCC cell N-glycome. VU-147 T is an HPV + cell line, while 2A3 is originally derived from FaDu cells and transfected with HPV type 16 E6 and E7 genes under control of the Moloney murine leukaemia virus (MoMuLV) promoter-enhancer sequence [28]. Interestingly, VU-147 T exhibited the highest level of complex-type N-glycans (35.6%), while the opposite was found for 2A3, which was the cell line with the lowest level of complex-type N-glycans (23.7%; Table 1). The high level of complex-type N-glycans found in VU-147 T is particularly due to the higher levels of tri antennary and larger, not further structurally defined N-glycans (Fig. 2).

While the overall level of complex-type N-glycans differed significantly between cell lines (Table 1), almost all these structures carried one or more sialic acid(s). Notably, a strong reduction in complex-type N-glycans was found in 2A3 cells, while their originator cell line (FaDu) showed essentially similar levels to, e.g. VU-147 T cells (33.9%; Table 1). It remains unclear if that is the consequence of insertion of HPV type 16 E6 and E7 genes or a secondary, off-target effect. Such off-target effects impacting cellular glycosylation as a consequence of gene-editing have been reported earlier in ovarian cancer cells [29]. CRISPR-*Cas9*-mediated disruption of *B3GNT5*, a key transferase in the (neo-) lacto series glycosphingolipid biosynthesis, led to the unexpected depletion of α2-6 sialylated N-glycans due to the lack of *ST6GAL1* expression in the ∆B3GNT5 cells [29]. It is conceivable that insertion of the E6 and E7 genes in combination with the neomycin resistance, which is associated with the used vector, led to a modulation of the glycosylation features now observed for the 2A3 cell line that differs significantly from the parental FaDu cells (Fig. 1B).

### Sialylation is a major feature of complex-type N-glycans in HNSCC cells

**Fig. 3:**
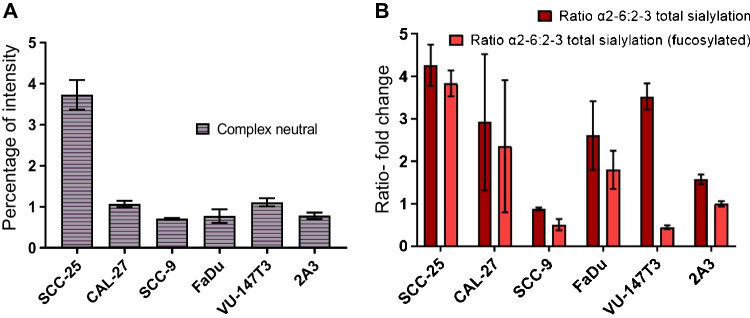
Sialylation is a major feature of complex-type N-glycans in HNSCC cells. Quantitative distribution of NeuAc-linked glycan in the complex-type glycans. **A** Quantitation of complex neutral glycans. **B** Ratio of α2-6 vs α2-3 sialylated glycans and sialylation with fucosylation

With exception of SCC-25, where slightly higher levels of neutral complex-type N-glycans were identified (3.7%), the levels of neutral, complex-type N-glycans were around 1% or lower in all other cell lines (Fig. 3A). Thus, sialylation can be considered to be a major feature of complex N-glycans in all analysed HNSCC cell lines (Table 1). To better understand the type of N-acetylneuraminic acid (NeuAc) linkages across the different cell lines, we used PGC-LC glycomics approach that allows for an easy differentiation of NeuAc linkages on N-glycans [30, 31]. While SCC-9 showed an almost balanced ratio between α2-6 and α2-3 linked N-glycans (Fig. 3B), the levels of α2-6 linked NeuAc was up to four times the one of α2-3 linked NeuAc in all other cell lines (Fig. 3B). These higher levels of α2-6 linked NeuAc was largely independent of core-fucosylation except in VU-147 T, where non-core fucosylated N-glycans were four times more likely to carry α2-6 linked NeuAc, while core-fucosylated ones exhibited almost equal levels of α2-3 and α2-6 NeuAc (Fig. 3B). Interestingly, a similar link between NeuAc linkage and core-fucosylation has been previously observed for glycoproteins obtained from non-melanoma skin cancer biopsies [32], hinting towards some form of higher-level connection between core-fucosylation and sialylation linkage.

### Core fucosylation is the major form of fucosylation in HNSCC N-glycans

**Fig. 4:**
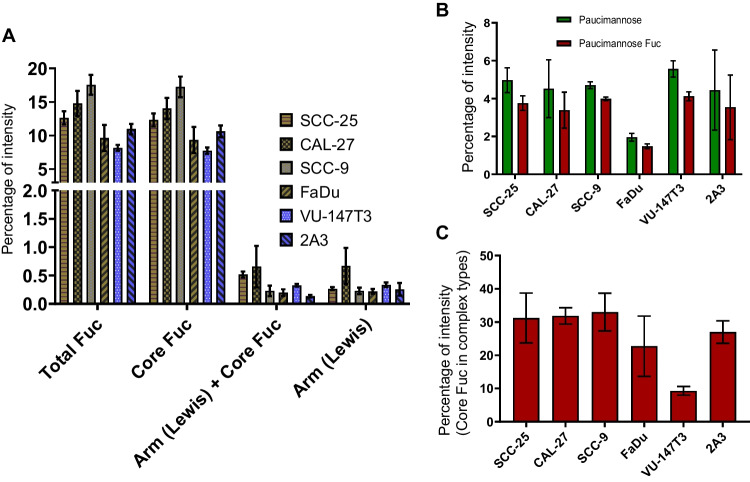
Core fucosylation is the major form of fucosylation in HNSCC N-glycan. Distribution of fucosylated-type glycans. **A** Proportion of different categories of fucosylated types of glycan. **B** Fucosylation in paucimannosidic glycans. **C** Core fucose in complex-type glycan category

Fucose plays a major role as a component of cancer-associated glyco-epitopes such as (but not limited to) Lewis X or Lewis Y [10, 33]. Core fucose was the major form of fucosylation found across all analysed HNSCC cell lines, ranging from 8 (VU-147 T) to 18% of all N-glycans (SCC-9). Less than 1% of N-glycans carried Lewis-type fucose residues (Fig. 4A), making this just a very minor proportion of glyco-epitopes in HNSCC cells. The levels of core-fucosylation, however, were distinctly different between cell lines and glycan-types.

Almost all paucimannose-type N-glycans were core-fucosylated (Fig. 4B). In Cal-27, SCC-9 and SCC-25, about one-third of all complex N-glycans carried a core-fucose. These levels were slightly lower in FaDu and 2A3 cells (around a quarter of all complex ones), but significantly lower in VU-147 T cells, where in average just one tenth of all complex N-glycans was core fucosylated (Fig. 4C). This clearly indicates towards a reduced expression of FUT8 in VU-147 T. While it is impossible to speculate about the cause for the indicated lower transcript or protein expression (or fucosyltransferase activity) of FUT8 in VU-147 T, the different origins of these cells (floor of mouth) might be a possible explanation as essentially all other cells are derived from epithelial origins (Supplementary Table 1).

### Phosphorylated oligomannose N-glycan levels vary across cell lines

**Fig. 5:**
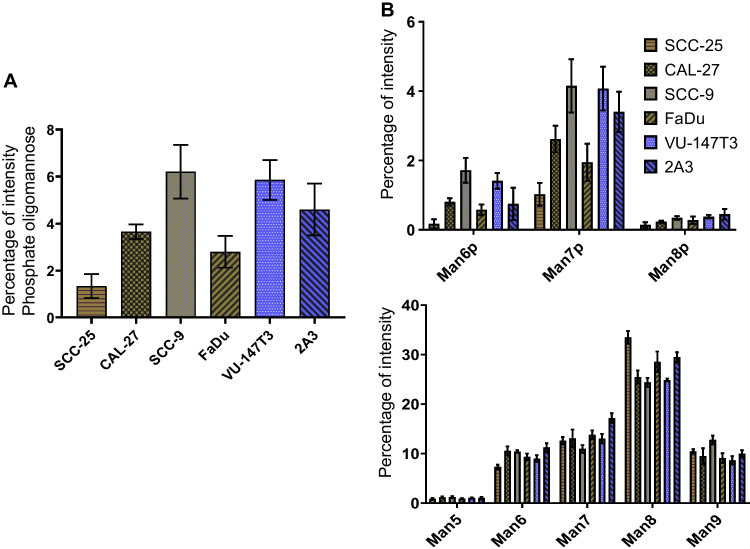
Phosphorylated oligomannose N-glycan levels vary across cell lines. Dissecting oligomannose-type glycan. **A** Quantitation of phosphate attached oligomannose glyacns. **B** Quantitative distribution of different categories of oligomannose types across the cell lines. Man6p = Mannose 6 phosphate

Mannose 6-phosphate (Man6P) is a common modification important to guide glycoproteins towards the lysosome via the Man-6-P receptor (M6PR) [34]. Thus, the levels of Man6P containing N-glycans could indicate towards an increased activity of lysosome. SCC-9 cells as well as the HPV + cell lines VU-147 T and 2A3 contained significantly higher levels of Man6P oligomannose-type N-glycans (up to 6% of the total N-glycan pool) compared to the other three cell lines that were between 1.5 and 3% (Fig. 5A). Independent of the overall level, all cell lines shared the same pattern that Man7 was by far the most abundant phosphorylated oligomannose N-glycan modified with up to two phosphate residues, while phosphate attached to Man8 and Man6 was detected in far lower amounts (Fig. 5B). Given that Man8 was clearly the most abundant oligomannose structure across all cell lines, this clearly indicates that for lysosomal glycoproteins Man7 carrying one or two phosphate residues appears to be the major N-glycan involved in lysosomal targeting by the M6PR (Fig. 5B).

### Using glycomics to dissect glycosyltransferase specificity: FUT8 core-fucosylates a variety of oligomannose-type N-glycans

**Fig. 6:**
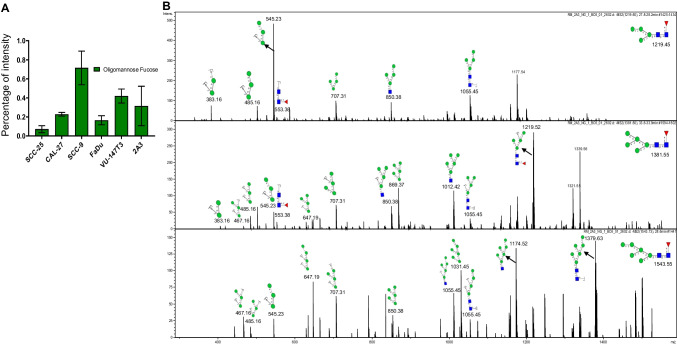
Core-fucosylation of oligomannose-type N-glycans. **A** Quantitation of core fucose attached oligomannose glycans. **B** Tandem MS data to identify oligomannose core-fucose structures

The substrate specificities of glycosyltransferases have traditionally been investigated in highly defined conditions that hardly can be considered to mimic the complex environment in which they have to act within the cell [35]. Core fucosylated oligomannose-type N-glycans have previously been reported in *MGAT1*-deficient (Lec1) Chinese hamster ovary (CHO) [36] and HEK293S cells [37], as well as in otherwise genetically unmodified porcine islet cells [38] and on human placental arylsulfatase A (though not confirmed by any MS2 fragmentation data) [39]. Yang et al. also identified that attachment of the N-glycan to a peptide/protein was a prerequisite for FUT8 to transfer a core-fucose onto an oligomannose-type N-glycan even in the absence of a GlcNAc on the α1-3 arm of the core mannose, while free oligomannose N-glycans remained unmodified by FUT8 in vitro [40]. In the analysed HNSCC cells, we found that core-fucose was attached to on Man4, Man5 and Man6 in up to 0.8% of all N-glycans (in SCC-9), while the levels of these N-glycans were less than 0.1% in SCC-25 (Fig. 6A, Supplementary Table 2 and Supplementary Table 5). Next to SCC-9, both HPV expressing cell lines (VU-147 T and 2A3) showed slightly higher levels of these core fucosylated, oligomannose-type N-glycans, while the levels in the remaining two (CAL-27 and FaDu) were around 0.2% or lower (Fig. 6A). To the best of our knowledge, these data for the first time confirm by tandem MS data that *MGAT1*-independent core fucosylation on oligomannose-type N-glycans can occur in otherwise unmodified human cells in low levels (Fig. 6B).

## Discussion

Despite the increasing prevalence of HNSCC and its known association with HPV infection, the N-glycome of the cell lines most widely used in HNSCC research has not been studied. For the first time, we demonstrated that while these cell lines exhibit similar general profile, significant quantitative differences in their N-glycosylation features exist that make each of them unique research resources (Fig. 1B). As reported in comparable studies for colon cancer [41] or breast cancer cell lines [42], oligomannose-type N-glycans were the most prevalent N-glycans in HNSCC cells. The levels of complex-type N-glycans ranged between 24 and 36% (Fig. 1B), with most of them being sialylated (Table 1). Interestingly, the levels of paucimannose N-glycans, which were found to be a signature of many human cancer types [43], were low and below 5% except in VU-147 T cells (Fig. 1B). The levels of phosphorylated oligomannose-type N-glycans, which are associated with lysosomal glycoproteins, were significantly higher just in SCC-9 and VU-147 T cells (≈6%, Fig. 5A). This could indicate differences in the levels (and thus likely also activity) of lysosomal degradation pathways. The same two cell lines also exhibited the highest levels of core-fucosylated oligomannose-type N-glycans (Fig. 6A), despite the fact that these cell lines also showed comparably high levels of complex-type N-glycans (30.5% and 35.6%, respectively). Attachment of the N-glycan to a protein is a known prerequisite for FUT8 to transfer a fucose onto an oligomannose N-glycan [40], and the fact that these were highest in cell lines that also exhibited high levels of complex-type N-glycans could indicate that this modification is just restricted to specific glycoproteins.

Sialylation of tumour tissues has been correlated with cancer progression, metastatic spreading and poor prognosis across many different cancer types [44, 45, 46]. Most complex-type N-glycans in the analysed HNSCC cell lines carried at least one sialic acid, with α2-6 linked NeuAc residues being the dominant form of sialylation in all cell lines except SCC-9, where almost equal levels of α2-3 and α2-6 linked NeuAc residues were observed (Table 1, Fig. 3B). This could impact recognition of these HNSCC cell surface glycoproteins by Galectins, given that α2-3 NeuAc carrying LacNAc epitopes can be recognised by Galectin 1 and 3, while the α2-6 NeuAc capping blocks this recognition [47]. In oral squamous cell carcinoma (OSCC), inhibition of Galectin-3 has been shown to overcome cetuximab‑resistance in murine animal models [48]. Yin et al. showed that in cetuximab‑resistant OSCC tumours, increased expression of Galectin‑3, p‑ERK1/2 and p‑Akt was observed. The use of a Gal‑3 inhibitor decreased the proliferation and invasion, while increasing the apoptosis of cetuximab‑resistant HSC3 cells. These data clearly demonstrate an intrinsic role of these cell surface glycoconjugates and their interactions within the tumour microenvironment in immunotherapy.

The interplay between the different sialyltransferases known to add NeuAc residues onto N-glycans, such as ST6GAL1 or the ST3GAL4/5/6, clearly plays a major role in this context of the tumour microenvironment. The inhibition of α2-3 NeuAc expression has been demonstrated to suppress the migration and metastasis in melanoma cells [49]. ST6GAL1 has been reported earlier to be associated which enhanced growth, survival and metastasis in multiple cancers (including pancreatic, prostate, breast and ovarian cancer) [50]. Increased α2-6 NeuAc levels on the human epidermal growth factor receptor 2 (HER2) have been reported to facilitate gastric cancer progression and resistance via activation of the Akt and ERK pathways [51]. While there is some information about Galectin expression levels in head and neck and thyroid carcinomas [52, 53], their specific role, interaction partners and contributions to HNSCC pathogenesis and precision treatment remains still unknown.

This study provides for the first time a comprehensive mapping of the N-glycome from in six widely used HNSCC cell lines. Our data constitute the basis for further studies aiming at better understanding how changes in the HNSCC glycome may contribute to the pathogenesis of these highly heterogenous cancers. Moreover, identification of HNSCC-specific glycome modifications may be exploited to improve the predictive and prognostic definition of these patients and provide novel targets for improved treatments.

## Supplementary Information

Below is the link to the electronic supplementary material.

Supplementary file1 (DOCX 1.39 MB)

Supplementary file2 (XLSX 1527 KB)
