# Supplementary material for: Head and neck cancer N-glycome traits are cell line and HPV status–dependent
Source: Anal Bioanal Chem. 2022 Oct 27;414(29-30):8401–11. doi: 10.1007/s00216-022-04376-x (PMC9712327; doi:10.1007/s00216-022-04376-x)
Supplement: Supplementary file 1 — Supplementary file1 (DOCX 1.39 MB) [file 216_2022_4376_MOESM1_ESM.docx]

**Supplementary Information**

**Head and Neck Cancer N-glycome traits are cell line and HPV-status dependent**

Mohammad Rasheduzzaman^1,2^, Abarna V. M. Murugan^3^, Xi Zhang^2^, Tiago Oliveira^3^, Riccardo Dolcetti^4,5,6,7^, Liz Kenny^8^, Newell W. Johnson^9^, Daniel Kolarich^3,10^*, Chamindie Punyadeera^1,2,9^*

1. Centre for Biomedical Technology, School of Biomedical Sciences, Faculty of Health, Queensland University of Technology, Kelvin Grove, QLD, Australia

2. Saliva and Liquid Biopsy Translational Laboratory, Griffith Institute for Drug Discovery, Griffith University, QLD, Australia

3. Institute for Glycomics, Griffith University, Gold Coast, QLD, Australia

4. Peter MacCallum Cancer Centre, University of Melbourne, Australia

5. Sir Peter MacCallum Department of Oncology, The University of Melbourne, Victoria 3010, Australia

6. Department of Microbiology and Immunology, The University of Melbourne, Victoria 3010, Australia

7. The University of Queensland Diamantina Institute, Brisbane, QLD, Australia

8. Department of Radiation Oncology, Cancer Care Services, Royal Brisbane and Women's Hospital, Joyce Tweddell Building, Herston, QLD, 4029, Australia

9. Menzies Health Institute Queensland, Griffith University, Gold Coast, Queensland, Australia

10. ARC Centre of Excellence for Nanoscale BioPhotonics, Griffith University, QLD, Australia

*Corresponding Authors

Professor Chamindie Punyadeera

Saliva and liquid biopsy translational laboratory, Griffith Institute for Drug Discovery, Griffith University, QLD, Australia

Telephone: +61 7 3138 0830 Email: c.punyadeera@griffith.edu.au

Associate Professor Daniel Kolarich
Institute for Glycomics, Griffith University, Gold Coast Campus, 4222 Southport, Queensland, Australia

Tel.: +61-7-5552 7026 Email: d.kolarich@griffith.edu.au

#### N-glycan release

Glycoproteins were precipitated using ice cold (-20°C) acetone. The resultant protein pellet was allowed to air dry at room temperature. The sample was resuspended using 8 M urea buffer by intensive vortexing. 50 µg of (glyco)proteins were immobilized on 0.45 µm pore size PVDF, washed and stained with Direct blue 71. Sample spots were excised and transferred into a flat bottom polypropylene 96‐well plate with 100 μL of 1% (wt/vol) PVP40 solution in 50% methanol and subsequently washed with LC-MS grade water. For each well, N‑glycans were released using 1 U PNGase F per 20 µg protein in 10 µL water and incubated overnight at 37°C [1]. The released N‐glycans were collected into fresh tubes and the wells washed twice with 20 μL of water. The washes were pooled to the respective sample tube and dried using the speedVac concentrator. Dried N-glycans were then reduced for 3 h at 50°C using 20 μL of 1 M NaBH_4_ in 50 mM KOH. Glacial acetic acid (1 μL) was used to neutralize the reduction reaction.

Reduced N-glycans were desalted as described previously [2], before being submitted to PGC-LC-ESI MS/MS glycomics analysis. Briefly, methanol washed AG 50W X8 cation exchange resin was packed on top of a ZipTip C18 tip. The prepared spin columns were washed and conditioned using 1 M HCl, methanol and water. Samples were then loaded to the respective columns and collected into fresh tubes. The desalting columns were washed twice with water to reduce losses. Desalted N-glycans were then dried under vacuum using a speedVac concentrator. Resulting borate salts were removed by washing at least three times with 100 µL methanol and subsequent drying of the samples. This process was repeated if any residual borate was visible [1,2].

The reduced N-glycans were further purified by porous-graphitized carbon (PGC) offline chromatography packed as microcolumns on top of C18 Zip Tips. The N‐glycans were eluted from the PGC columns using 0.1% trifluoroacetic acid (TFA) in 50% acetonitrile (ACN) (v/v). The eluted samples were dried and reconstituted in 50 µL water, centrifuged at 14,000 x *g* for 10 min at 4°C, and the supernatant finally transferred into high recovery glass vials (water) for LC‐MS/MS analysis. N-glycans obtained from bovine fetuin (Sigma) were released using the sample protocol and included as controls.

#### PGC-LC-ESI MS/MS

PGC-nano LC ESI-MS/MS glycomics was executed with an amaZon speed ion trap (IT-MS, Bruker, Bremen, Germany) coupled to an Ultimate 3000 UHPLC system (Dionex/Thermo). The amaZon IT-MS was equipped with a CaptiveSpray ion Source (Bruker, Bremen, Germany). The MS instrument was run in negative ion mode. The collision induced dissociation (CID) fragmentation was performed on the five most intense precursors of each MS scan. An *m/z* range 300–1,800 was settled for data-dependent precursor scanning. MS scan was performed on Ultra Scan mode and MS/MS scanning was performed using the Enhanced Resolution mode. 2μg of Glycan sample was injected through the autosampler and separated using an analytical PGC column (Hypercarb™ PGC Column, New Objectives, 150 mm × 100 μm, 5 μm particle size, maintained at 45°C). 10 mM ammonium bicarbonate (solvent A) and 70% ACN in 10 mM ammonium bicarbonate (solvent B) were used as the mobile phases for glycan elution. The flow rate executed for loading was 0.5 μL/min. Valve switch was settled at 40.4 min. The flow rate used to separate the N glycans was 0.5 μL/min. The gradient system used to separate the N-glycan as follow: 0-41.9 min, 0% B; 42-43 min, linear increase to 10% B; 43-105 min, linear increase to 30% B. Washing and re-equilibration steps were performed: 105-107 min, linear increase to 90% B; 107-112 min, held at 90% B; 112-113 min, return to 0% B; 113-145 min, re-equilibration at 0% B. The second valve switch was fixed to 75 min. Eluted glycans were then ionised using ESI in negative ion mode with an ion trap mass analyser. Capillary voltage was +1.3 kv, nitrogen drying gas flow 3 L/min at 85 °C, [2].

#### Determination of glycan structures and quantitation of relative abundances

Glycan data generated from the PGC-LC-MS/MS was processed by the ESI-Compass DataAnalysis 4.2 software, Bruker). GlycoMod ([**http://www.expasy.ch/tools/glycomod**](http://www.expasy.ch/tools/glycomod)) was used to identify possible N-glycan structures. N-glycan structures were detected by manual spectral screening, annotation, isomeric structural elucidation, PGC retention time, negative-ion mode fragmentation behaviour [3], and biosynthesis pathways [4,5]. GlycoWorkBench v2.1 software was used for manual structural depiction of glycan structures and to annotate of MS2 fragment spectra [6].

Representative MS/MS spectra for each identified glycan structure (if available) can be generated from the information given (m/z, intensity). Mass lists of fragment spectra were created from raw data using DataAnalysis Version 4.2 (Bruker) with an absolute intensity threshold of 50 (no rel. threshold used), employed S/N ratio of 1 and a peak width (FWHM (*m/z*)) of 0.1.

Relative abundances of glycan structures were identified utilizing the area under the curve (AUC) from the respective individual chromatographic traces (extracted ion chromatograms, EIC) of the corresponding monoisotopic precursors using Skyline software (https://skyline.ms/project/home/software/Skyline/begin.view). The integration limits were manually checked for each compound. The sum of all identified and quantified AUCs of the glycan structures within a sample was defined as 100% and the individual relative abundances were measured from the corresponding AUC values (Supplementary table 2). Experimental workflow for Glycan profiling is depicted in the supplementary figure 1.


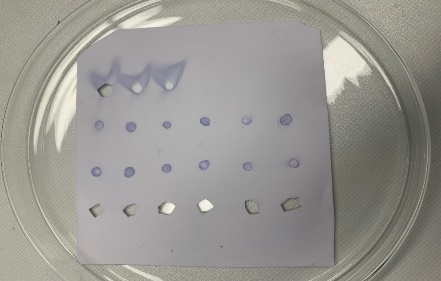


6. LC-MS/MS glycomics analyses

7. Data processing and interpretation

1. Cells harvested (80-85% confluence)

2. Dot blotting (overnight)

3. PNGaseF digestion (12H) for release of N-glycans from proteins

Reduction and desalting

4. Sample injection into HPLC

5. Mass spectrometry


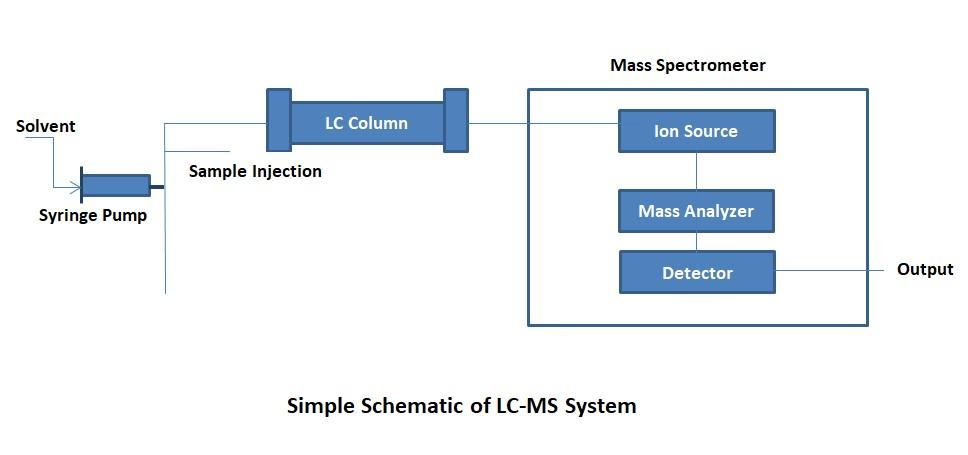

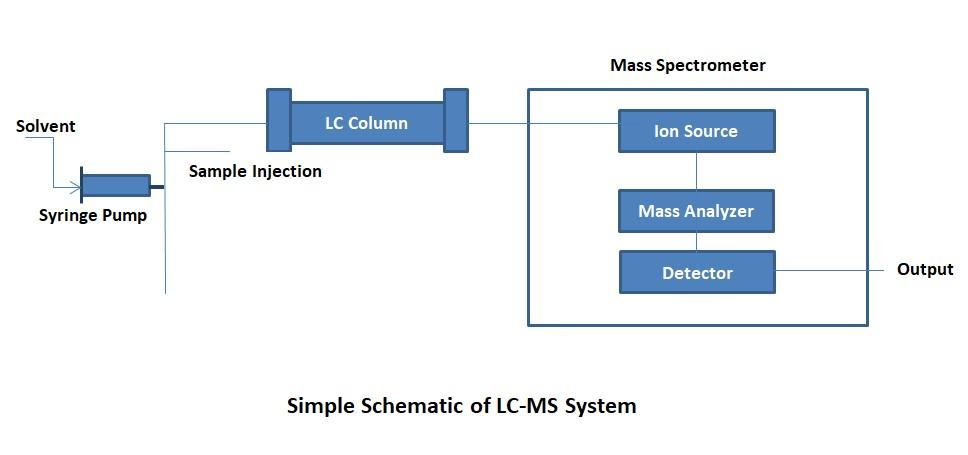

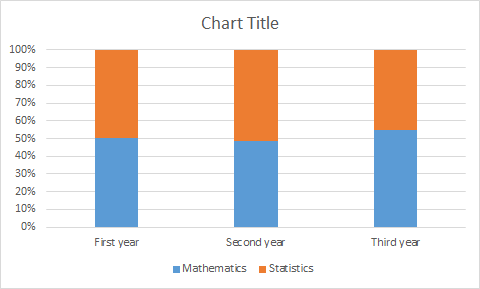

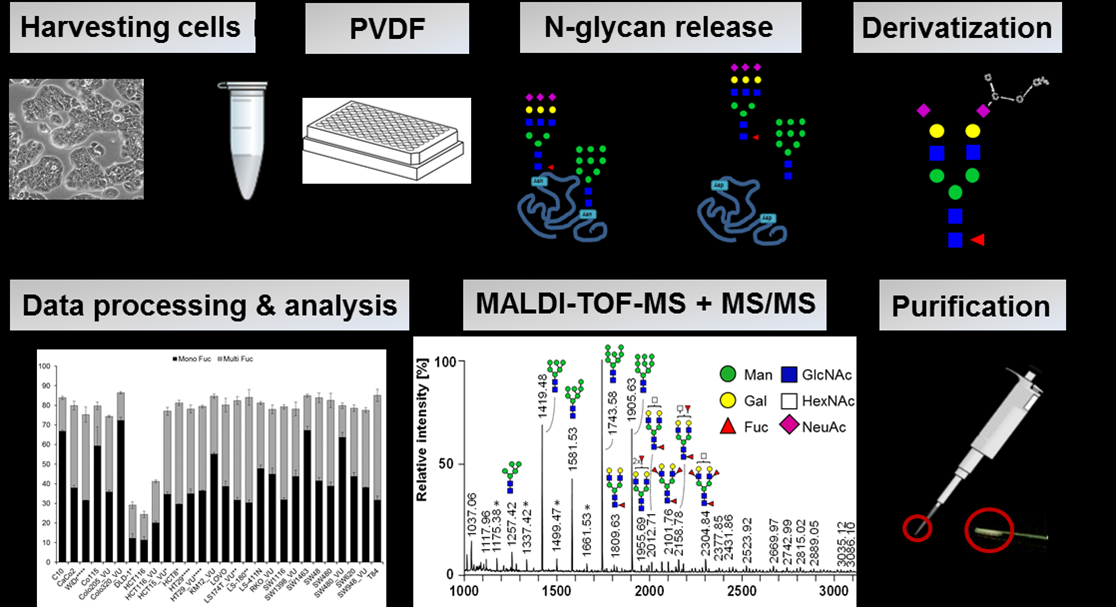

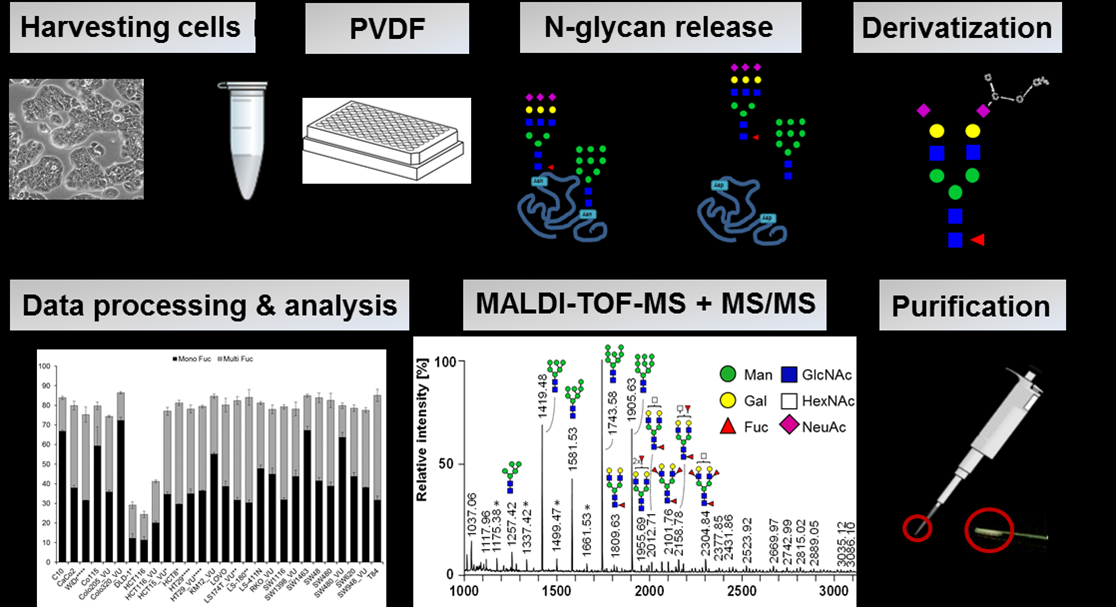


Supplementary Figure 1: Workflow for glycomics profiling

Supplementary Table 1: HNSCC cell lines information

| **Name** | **Origin (Human-subsite not known)** | **GMO** | **Sources** | **Others** |
| --- | --- | --- | --- | --- |
| **FaDu (n=6)** | HNSCC (pharynx) | No | ATCC  (STR profiled) | Epithelial type small rounded shape, collected from 56 years old male. Squamous cell carcinoma |
| **SCC-25 (n=6)** | HNSCC (tongue) | No | ATCC (STR profiled) | Epithelial type small rounded shape, collected from 70 years old male. Squamous cell carcinoma |
| **Cal-27 (n=6)** | HNSCC (tongue) | No | ATCC (STR profiled) | Epithelial type small rounded shape, collected from 56 years old Caucasian male. Squamous cell carcinoma |
| **SCC-9 (n=4)** | HNSCC (tongue) | No | ATCC (STR profiled) | 25 years old male, small rounded shape, Squamous cell carcinoma |
| **2A3 (n=6)** | Biologically similar to FaDu except HNSCC (HPV+) gene E6/E7 transfected (pharynx) | Yes | ATCC (STR profiled) | from 56 years old male: Epithelial like small rounded shape, collected from 56 years old male. Squamous cell carcinoma |
| **VU-147T (n=6)** | HNSCC (HPV+) (pharynx) | No | Generous gift | 58 years old male: floor of mouth squamous cell carcinoma. Squamous cell carcinoma of the oral cavity. Collected from 58 years old male |

Supplementary Table 9: Standard Deviation of Figure 1B

| Traits | VU-147T | SCC-25 | CAL-27 | SCC-9 | FaDu | 2A3 |
| --- | --- | --- | --- | --- | --- | --- |
| Paucimannose | 0.43 | 0.66 | 1.53 | 0.17 | 0.20 | 2.11 |
| Oligomannose | 2.19 | 2.26 | 2.38 | 0.88 | 3.06 | 0.66 |
| Hybrid | 0.18 | 0.04 | 0.61 | 0.09 | 0.11 | 0.38 |
| Complex | 2.21 | 1.94 | 0.86 | 0.88 | 3.06 | 1.47 |

| **Statistical analysis: 2way ANOVA report**  **Figure 1B** |  |  |  |  |  |
| --- | --- | --- | --- | --- | --- |
| Tukey's multiple comparisons test | Predicted (LS) mean diff. | 95.00% CI of diff. | Significant | Summary | Adjusted P Value |
|  |  |  |  |  |  |
| Paucimannose |  |  |  |  |  |
| SCC-25 vs. CAL-27 | 0.4500 | -2.180 to 3.080 | No | ns | 0.9962 |
| SCC-25 vs. SCC-9 | 0.2633 | -2.678 to 3.204 | No | ns | 0.9998 |
| SCC-25 vs. FaDu | 3.018 | 0.3880 to 5.649 | Yes | * | 0.0147 |
| SCC-25 vs. VU-147T3 | -0.5933 | -3.224 to 2.037 | No | ns | 0.9864 |
| SCC-25 vs. 2A3 | 0.5250 | -2.105 to 3.155 | No | ns | 0.9922 |
| CAL-27 vs. SCC-9 | -0.1867 | -3.128 to 2.754 | No | ns | >0.9999 |
| CAL-27 vs. FaDu | 2.568 | -0.06205 to 5.199 | No | ns | 0.0598 |
| CAL-27 vs. VU-147T3 | -1.043 | -3.674 to 1.587 | No | ns | 0.8591 |
| CAL-27 vs. 2A3 | 0.07500 | -2.555 to 2.705 | No | ns | >0.9999 |
| SCC-9 vs. FaDu | 2.755 | -0.1859 to 5.696 | No | ns | 0.0800 |
| SCC-9 vs. VU-147T3 | -0.8567 | -3.798 to 2.084 | No | ns | 0.9584 |
| SCC-9 vs. 2A3 | 0.2617 | -2.679 to 3.203 | No | ns | 0.9998 |
| FaDu vs. VU-147T3 | -3.612 | -6.242 to -0.9813 | Yes | ** | 0.0017 |
| FaDu vs. 2A3 | -2.493 | -5.124 to 0.1370 | No | ns | 0.0738 |
| VU-147T3 vs. 2A3 | 1.118 | -1.512 to 3.749 | No | ns | 0.8198 |
|  |  |  |  |  |  |
| Oligomannose |  |  |  |  |  |
| SCC-25 vs. CAL-27 | 5.470 | 2.840 to 8.100 | Yes | **** | <0.0001 |
| SCC-25 vs. SCC-9 | 5.179 | 2.238 to 8.120 | Yes | **** | <0.0001 |
| SCC-25 vs. FaDu | 3.627 | 0.9963 to 6.257 | Yes | ** | 0.0016 |
| SCC-25 vs. VU-147T3 | 8.823 | 6.193 to 11.45 | Yes | **** | <0.0001 |
| SCC-25 vs. 2A3 | -3.917 | -6.547 to -1.286 | Yes | *** | 0.0005 |
| CAL-27 vs. SCC-9 | -0.2908 | -3.232 to 2.650 | No | ns | 0.9997 |
| CAL-27 vs. FaDu | -1.843 | -4.474 to 0.7870 | No | ns | 0.3311 |
| CAL-27 vs. VU-147T3 | 3.353 | 0.7230 to 5.984 | Yes | ** | 0.0045 |
| CAL-27 vs. 2A3 | -9.387 | -12.02 to -6.756 | Yes | **** | <0.0001 |
| SCC-9 vs. FaDu | -1.553 | -4.493 to 1.388 | No | ns | 0.6453 |
| SCC-9 vs. VU-147T3 | 3.644 | 0.7033 to 6.585 | Yes | ** | 0.0063 |
| SCC-9 vs. 2A3 | -9.096 | -12.04 to -6.155 | Yes | **** | <0.0001 |
| FaDu vs. VU-147T3 | 5.197 | 2.566 to 7.827 | Yes | **** | <0.0001 |
| FaDu vs. 2A3 | -7.543 | -10.17 to -4.913 | Yes | **** | <0.0001 |
| VU-147T3 vs. 2A3 | -12.74 | -15.37 to -10.11 | Yes | **** | <0.0001 |
|  |  |  |  |  |  |
| Complex |  |  |  |  |  |
| SCC-25 vs. CAL-27 | -4.700 | -7.330 to -2.070 | Yes | **** | <0.0001 |
| SCC-25 vs. SCC-9 | -2.703 | -5.644 to 0.2375 | No | ns | 0.0905 |
| SCC-25 vs. FaDu | -6.290 | -8.920 to -3.660 | Yes | **** | <0.0001 |
| SCC-25 vs. VU-147T3 | -8.018 | -10.65 to -5.388 | Yes | **** | <0.0001 |
| SCC-25 vs. 2A3 | 3.893 | 1.263 to 6.524 | Yes | *** | 0.0005 |
| CAL-27 vs. SCC-9 | 1.997 | -0.9442 to 4.938 | No | ns | 0.3668 |
| CAL-27 vs. FaDu | -1.590 | -4.220 to 1.040 | No | ns | 0.5003 |
| CAL-27 vs. VU-147T3 | -3.318 | -5.949 to -0.6880 | Yes | ** | 0.0051 |
| CAL-27 vs. 2A3 | 8.593 | 5.963 to 11.22 | Yes | **** | <0.0001 |
| SCC-9 vs. FaDu | -3.587 | -6.528 to -0.6458 | Yes | ** | 0.0076 |
| SCC-9 vs. VU-147T3 | -5.315 | -8.256 to -2.374 | Yes | **** | <0.0001 |
| SCC-9 vs. 2A3 | 6.597 | 3.656 to 9.538 | Yes | **** | <0.0001 |
| FaDu vs. VU-147T3 | -1.728 | -4.359 to 0.9020 | No | ns | 0.4044 |
| FaDu vs. 2A3 | 10.18 | 7.553 to 12.81 | Yes | **** | <0.0001 |
| VU-147T3 vs. 2A3 | 11.91 | 9.281 to 14.54 | Yes | **** | <0.0001 |
|  |  |  |  |  |  |
| Hybrid neutral |  |  |  |  |  |
| SCC-25 vs. CAL-27 | -1.218 | -3.849 to 1.412 | No | ns | 0.7604 |
| SCC-25 vs. SCC-9 | -2.741 | -5.682 to 0.2000 | No | ns | 0.0828 |
| SCC-25 vs. FaDu | -0.3550 | -2.985 to 2.275 | No | ns | 0.9988 |
| SCC-25 vs. VU-147T3 | -0.2100 | -2.840 to 2.420 | No | ns | >0.9999 |
| SCC-25 vs. 2A3 | -0.5033 | -3.134 to 2.127 | No | ns | 0.9936 |
| CAL-27 vs. SCC-9 | -1.523 | -4.463 to 1.418 | No | ns | 0.6642 |
| CAL-27 vs. FaDu | 0.8633 | -1.767 to 3.494 | No | ns | 0.9319 |
| CAL-27 vs. VU-147T3 | 1.008 | -1.622 to 3.639 | No | ns | 0.8757 |
| CAL-27 vs. 2A3 | 0.7150 | -1.915 to 3.345 | No | ns | 0.9690 |
| SCC-9 vs. FaDu | 2.386 | -0.5550 to 5.327 | No | ns | 0.1824 |
| SCC-9 vs. VU-147T3 | 2.531 | -0.4100 to 5.472 | No | ns | 0.1343 |
| SCC-9 vs. 2A3 | 2.238 | -0.7034 to 5.178 | No | ns | 0.2432 |
| FaDu vs. VU-147T3 | 0.1450 | -2.485 to 2.775 | No | ns | >0.9999 |
| FaDu vs. 2A3 | -0.1483 | -2.779 to 2.482 | No | ns | >0.9999 |
| VU-147T3 vs. 2A3 | -0.2933 | -2.924 to 2.337 | No | ns | 0.9995 |

**Figure 2**

| Bi antennary |  |  |  |  |  |
| --- | --- | --- | --- | --- | --- |
| SCC-25 vs. CAL-27 | -3.175 | -5.015 to -1.335 | Yes | **** | <0.0001 |
| SCC-25 vs. SCC-9 | -1.582 | -3.639 to 0.4754 | No | ns | 0.2298 |
| SCC-25 vs. FaDu | -2.777 | -4.617 to -0.9368 | Yes | *** | 0.0004 |
| SCC-25 vs. VU-147T3 | 2.640 | 0.8001 to 4.480 | Yes | *** | 0.0010 |
| SCC-25 vs. 2A3 | 3.940 | 2.100 to 5.780 | Yes | **** | <0.0001 |
| CAL-27 vs. SCC-9 | 1.593 | -0.4637 to 3.650 | No | ns | 0.2227 |
| CAL-27 vs. FaDu | 0.3983 | -1.442 to 2.238 | No | ns | 0.9883 |
| CAL-27 vs. VU-147T3 | 5.815 | 3.975 to 7.655 | Yes | **** | <0.0001 |
| CAL-27 vs. 2A3 | 7.115 | 5.275 to 8.955 | Yes | **** | <0.0001 |
| SCC-9 vs. FaDu | -1.195 | -3.252 to 0.8621 | No | ns | 0.5394 |
| SCC-9 vs. VU-147T3 | 4.222 | 2.165 to 6.279 | Yes | **** | <0.0001 |
| SCC-9 vs. 2A3 | 5.522 | 3.465 to 7.579 | Yes | **** | <0.0001 |
| FaDu vs. VU-147T3 | 5.417 | 3.577 to 7.257 | Yes | **** | <0.0001 |
| FaDu vs. 2A3 | 6.717 | 4.877 to 8.557 | Yes | **** | <0.0001 |
| VU-147T3 vs. 2A3 | 1.300 | -0.5399 to 3.140 | No | ns | 0.3179 |
|  |  |  |  |  |  |
| Tri antennary |  |  |  |  |  |
| SCC-25 vs. CAL-27 | 0.01167 | -1.828 to 1.852 | No | ns | >0.9999 |
| SCC-25 vs. SCC-9 | 0.1017 | -1.955 to 2.159 | No | ns | >0.9999 |
| SCC-25 vs. FaDu | -1.845 | -3.685 to -0.005105 | Yes | * | 0.0490 |
| SCC-25 vs. VU-147T3 | -3.380 | -5.220 to -1.540 | Yes | **** | <0.0001 |
| SCC-25 vs. 2A3 | 1.195 | -0.6449 to 3.035 | No | ns | 0.4129 |
| CAL-27 vs. SCC-9 | 0.09000 | -1.967 to 2.147 | No | ns | >0.9999 |
| CAL-27 vs. FaDu | -1.857 | -3.697 to -0.01677 | Yes | * | 0.0466 |
| CAL-27 vs. VU-147T3 | -3.392 | -5.232 to -1.552 | Yes | **** | <0.0001 |
| CAL-27 vs. 2A3 | 1.183 | -0.6566 to 3.023 | No | ns | 0.4241 |
| SCC-9 vs. FaDu | -1.947 | -4.004 to 0.1104 | No | ns | 0.0743 |
| SCC-9 vs. VU-147T3 | -3.482 | -5.539 to -1.425 | Yes | **** | <0.0001 |
| SCC-9 vs. 2A3 | 1.093 | -0.9637 to 3.150 | No | ns | 0.6332 |
| FaDu vs. VU-147T3 | -1.535 | -3.375 to 0.3049 | No | ns | 0.1568 |
| FaDu vs. 2A3 | 3.040 | 1.200 to 4.880 | Yes | **** | <0.0001 |
| VU-147T3 vs. 2A3 | 4.575 | 2.735 to 6.415 | Yes | **** | <0.0001 |
|  |  |  |  |  |  |
| ND |  |  |  |  |  |
| SCC-25 vs. CAL-27 | -1.398 | -3.238 to 0.4416 | No | ns | 0.2412 |
| SCC-25 vs. SCC-9 | -1.247 | -3.304 to 0.8104 | No | ns | 0.4920 |
| SCC-25 vs. FaDu | -1.832 | -3.672 to 0.008228 | No | ns | 0.0517 |
| SCC-25 vs. VU-147T3 | -6.808 | -8.648 to -4.968 | Yes | **** | <0.0001 |
| SCC-25 vs. 2A3 | -0.9100 | -2.750 to 0.9299 | No | ns | 0.7011 |
| CAL-27 vs. SCC-9 | 0.1517 | -1.905 to 2.209 | No | ns | >0.9999 |
| CAL-27 vs. FaDu | -0.4333 | -2.273 to 1.407 | No | ns | 0.9830 |
| CAL-27 vs. VU-147T3 | -5.410 | -7.250 to -3.570 | Yes | **** | <0.0001 |
| CAL-27 vs. 2A3 | 0.4883 | -1.352 to 2.328 | No | ns | 0.9712 |
| SCC-9 vs. FaDu | -0.5850 | -2.642 to 1.472 | No | ns | 0.9612 |
| SCC-9 vs. VU-147T3 | -5.562 | -7.619 to -3.505 | Yes | **** | <0.0001 |
| SCC-9 vs. 2A3 | 0.3367 | -1.720 to 2.394 | No | ns | 0.9968 |
| FaDu vs. VU-147T3 | -4.977 | -6.817 to -3.137 | Yes | **** | <0.0001 |
| FaDu vs. 2A3 | 0.9217 | -0.9182 to 2.762 | No | ns | 0.6897 |
| VU-147T3 vs. 2A3 | 5.898 | 4.058 to 7.738 | Yes | **** | <0.0001 |

**Figure 3A**

**Complex neutral**

| SCC-25 vs. CAL-27 | 2.660 | 2.050 to 3.270 | Yes | **** | <0.0001 |
| --- | --- | --- | --- | --- | --- |
| SCC-25 vs. SCC-9 | 3.021 | 2.031 to 4.010 | Yes | ** | 0.0021 |
| SCC-25 vs. FaDu | 2.955 | 2.550 to 3.360 | Yes | **** | <0.0001 |
| SCC-25 vs. VU-147T3 | 2.620 | 2.095 to 3.145 | Yes | **** | <0.0001 |
| SCC-25 vs. 2A3 | 2.948 | 2.407 to 3.490 | Yes | **** | <0.0001 |
| CAL-27 vs. SCC-9 | 0.3608 | 0.1368 to 0.5848 | Yes | * | 0.0132 |
| CAL-27 vs. FaDu | 0.2950 | -0.03281 to 0.6228 | No | ns | 0.0739 |
| CAL-27 vs. VU-147T3 | -0.04000 | -0.3009 to 0.2209 | No | ns | 0.9803 |
| CAL-27 vs. 2A3 | 0.2883 | 0.09025 to 0.4864 | Yes | * | 0.0107 |
| SCC-9 vs. FaDu | -0.06583 | -0.4850 to 0.3534 | No | ns | 0.9260 |
| SCC-9 vs. VU-147T3 | -0.4008 | -0.7234 to -0.07823 | Yes | * | 0.0275 |
| SCC-9 vs. 2A3 | -0.07250 | -0.2542 to 0.1092 | No | ns | 0.4032 |
| FaDu vs. VU-147T3 | -0.3350 | -0.5479 to -0.1221 | Yes | ** | 0.0076 |
| FaDu vs. 2A3 | -0.006667 | -0.2247 to 0.2113 | No | ns | >0.9999 |
| VU-147T3 vs. 2A3 | 0.3283 | 0.1601 to 0.4966 | Yes | ** | 0.0028 |

**Figure 3B**

| Ratio α2-6:2-3 total sialylation |  |  |  |  |  |
| --- | --- | --- | --- | --- | --- |
| SCC-25 vs. CAL-27 | 1.345 | 0.06384 to 2.626 | Yes | * | 0.0343 |
| SCC-25 vs. SCC-9 | 3.392 | 1.959 to 4.824 | Yes | **** | <0.0001 |
| SCC-25 vs. FaDu | 1.660 | 0.3792 to 2.942 | Yes | ** | 0.0043 |
| SCC-25 vs. VU-147T3 | 0.7389 | -0.5423 to 2.020 | No | ns | 0.5365 |
| SCC-25 vs. 2A3 | 2.689 | 1.408 to 3.970 | Yes | **** | <0.0001 |
| CAL-27 vs. SCC-9 | 2.047 | 0.6141 to 3.479 | Yes | ** | 0.0012 |
| CAL-27 vs. FaDu | 0.3154 | -0.9658 to 1.597 | No | ns | 0.9779 |
| CAL-27 vs. VU-147T3 | -0.6061 | -1.887 to 0.6750 | No | ns | 0.7292 |
| CAL-27 vs. 2A3 | 1.344 | 0.06304 to 2.625 | Yes | * | 0.0345 |
| SCC-9 vs. FaDu | -1.731 | -3.164 to -0.2988 | Yes | ** | 0.0093 |
| SCC-9 vs. VU-147T3 | -2.653 | -4.085 to -1.220 | Yes | **** | <0.0001 |
| SCC-9 vs. 2A3 | -0.7023 | -2.135 to 0.7301 | No | ns | 0.6987 |
| FaDu vs. VU-147T3 | -0.9215 | -2.203 to 0.3597 | No | ns | 0.2911 |
| FaDu vs. 2A3 | 1.029 | -0.2523 to 2.310 | No | ns | 0.1848 |
| VU-147T3 vs. 2A3 | 1.950 | 0.6692 to 3.231 | Yes | *** | 0.0005 |
|  |  |  |  |  |  |
| Ratio α2-6:2-3 total sialylation (fucosylated) |  |  |  |  |  |
| SCC-25 vs. CAL-27 | 1.483 | 0.2022 to 2.764 | Yes | * | 0.0144 |
| SCC-25 vs. SCC-9 | 3.333 | 1.900 to 4.765 | Yes | **** | <0.0001 |
| SCC-25 vs. FaDu | 2.040 | 0.7588 to 3.321 | Yes | *** | 0.0002 |
| SCC-25 vs. VU-147T3 | 3.393 | 2.112 to 4.674 | Yes | **** | <0.0001 |
| SCC-25 vs. 2A3 | 2.840 | 1.559 to 4.121 | Yes | **** | <0.0001 |
| CAL-27 vs. SCC-9 | 1.849 | 0.4168 to 3.282 | Yes | ** | 0.0045 |
| CAL-27 vs. FaDu | 0.5567 | -0.7245 to 1.838 | No | ns | 0.7934 |
| CAL-27 vs. VU-147T3 | 1.910 | 0.6288 to 3.191 | Yes | *** | 0.0007 |
| CAL-27 vs. 2A3 | 1.357 | 0.07551 to 2.638 | Yes | * | 0.0320 |
| SCC-9 vs. FaDu | -1.293 | -2.725 to 0.1399 | No | ns | 0.0994 |
| SCC-9 vs. VU-147T3 | 0.06083 | -1.372 to 1.493 | No | ns | >0.9999 |
| SCC-9 vs. 2A3 | -0.4925 | -1.925 to 0.9399 | No | ns | 0.9112 |
| FaDu vs. VU-147T3 | 1.353 | 0.07217 to 2.634 | Yes | * | 0.0326 |
| FaDu vs. 2A3 | 0.8000 | -0.4812 to 2.081 | No | ns | 0.4477 |
| VU-147T3 vs. 2A3 | -0.5533 | -1.834 to 0.7278 | No | ns | 0.7975 |

| **Figure 4A**  Total Fucose |  |  |  |  |  |
| --- | --- | --- | --- | --- | --- |
| SCC-25 vs. CAL-27 | -2.142 | -3.732 to -0.5510 | Yes | ** | 0.0022 |
| SCC-25 vs. SCC-9 | -4.925 | -6.703 to -3.147 | Yes | **** | <0.0001 |
| SCC-25 vs. FaDu | 3.010 | 1.419 to 4.601 | Yes | **** | <0.0001 |
| SCC-25 vs. VU-147T3 | 4.468 | 2.878 to 6.059 | Yes | **** | <0.0001 |
| SCC-25 vs. 2A3 | 1.667 | 0.07602 to 3.257 | Yes | * | 0.0343 |
| CAL-27 vs. SCC-9 | -2.783 | -4.562 to -1.005 | Yes | *** | 0.0002 |
| CAL-27 vs. FaDu | 5.152 | 3.561 to 6.742 | Yes | **** | <0.0001 |
| CAL-27 vs. VU-147T3 | 6.610 | 5.019 to 8.201 | Yes | **** | <0.0001 |
| CAL-27 vs. 2A3 | 3.808 | 2.218 to 5.399 | Yes | **** | <0.0001 |
| SCC-9 vs. FaDu | 7.935 | 6.157 to 9.713 | Yes | **** | <0.0001 |
| SCC-9 vs. VU-147T3 | 9.393 | 7.615 to 11.17 | Yes | **** | <0.0001 |
| SCC-9 vs. 2A3 | 6.592 | 4.813 to 8.370 | Yes | **** | <0.0001 |
| FaDu vs. VU-147T3 | 1.458 | -0.1323 to 3.049 | No | ns | 0.0921 |
| FaDu vs. 2A3 | -1.343 | -2.934 to 0.2473 | No | ns | 0.1488 |
| VU-147T3 vs. 2A3 | -2.802 | -4.392 to -1.211 | Yes | **** | <0.0001 |
|  |  |  |  |  |  |
| Core Fucose |  |  |  |  |  |
| SCC-25 vs. CAL-27 | -1.708 | -3.299 to -0.1177 | Yes | * | 0.0276 |
| SCC-25 vs. SCC-9 | -4.915 | -6.693 to -3.137 | Yes | **** | <0.0001 |
| SCC-25 vs. FaDu | 2.982 | 1.391 to 4.572 | Yes | **** | <0.0001 |
| SCC-25 vs. VU-147T3 | 4.573 | 2.983 to 6.164 | Yes | **** | <0.0001 |
| SCC-25 vs. 2A3 | 1.695 | 0.1044 to 3.286 | Yes | * | 0.0296 |
| CAL-27 vs. SCC-9 | -3.207 | -4.985 to -1.428 | Yes | **** | <0.0001 |
| CAL-27 vs. FaDu | 4.690 | 3.099 to 6.281 | Yes | **** | <0.0001 |
| CAL-27 vs. VU-147T3 | 6.282 | 4.691 to 7.872 | Yes | **** | <0.0001 |
| CAL-27 vs. 2A3 | 3.403 | 1.813 to 4.994 | Yes | **** | <0.0001 |
| SCC-9 vs. FaDu | 7.897 | 6.118 to 9.675 | Yes | **** | <0.0001 |
| SCC-9 vs. VU-147T3 | 9.488 | 7.710 to 11.27 | Yes | **** | <0.0001 |
| SCC-9 vs. 2A3 | 6.610 | 4.832 to 8.388 | Yes | **** | <0.0001 |
| FaDu vs. VU-147T3 | 1.592 | 0.001017 to 3.182 | Yes | * | 0.0498 |
| FaDu vs. 2A3 | -1.287 | -2.877 to 0.3040 | No | ns | 0.1850 |
| VU-147T3 vs. 2A3 | -2.878 | -4.469 to -1.288 | Yes | **** | <0.0001 |
|  |  |  |  |  |  |
| Arm + Core fuco (Lewis) |  |  |  |  |  |
| SCC-25 vs. CAL-27 | -0.1400 | -1.731 to 1.451 | No | ns | 0.9998 |
| SCC-25 vs. SCC-9 | 0.2892 | -1.489 to 2.068 | No | ns | 0.9970 |
| SCC-25 vs. FaDu | 0.3183 | -1.272 to 1.909 | No | ns | 0.9921 |
| SCC-25 vs. VU-147T3 | 0.1900 | -1.401 to 1.781 | No | ns | 0.9993 |
| SCC-25 vs. 2A3 | 0.3817 | -1.209 to 1.972 | No | ns | 0.9821 |
| CAL-27 vs. SCC-9 | 0.4292 | -1.349 to 2.208 | No | ns | 0.9816 |
| CAL-27 vs. FaDu | 0.4583 | -1.132 to 2.049 | No | ns | 0.9602 |
| CAL-27 vs. VU-147T3 | 0.3300 | -1.261 to 1.921 | No | ns | 0.9907 |
| CAL-27 vs. 2A3 | 0.5217 | -1.069 to 2.112 | No | ns | 0.9321 |
| SCC-9 vs. FaDu | 0.02917 | -1.749 to 1.808 | No | ns | >0.9999 |
| SCC-9 vs. VU-147T3 | -0.09917 | -1.878 to 1.679 | No | ns | >0.9999 |
| SCC-9 vs. 2A3 | 0.09250 | -1.686 to 1.871 | No | ns | >0.9999 |
| FaDu vs. VU-147T3 | -0.1283 | -1.719 to 1.462 | No | ns | >0.9999 |
| FaDu vs. 2A3 | 0.06333 | -1.527 to 1.654 | No | ns | >0.9999 |
| VU-147T3 vs. 2A3 | 0.1917 | -1.399 to 1.782 | No | ns | 0.9993 |
|  |  |  |  |  |  |
| Arm (Lewis) |  |  |  |  |  |
| SCC-25 vs. CAL-27 | -0.4017 | -1.992 to 1.189 | No | ns | 0.9775 |
| SCC-25 vs. SCC-9 | 0.03750 | -1.741 to 1.816 | No | ns | >0.9999 |
| SCC-25 vs. FaDu | 0.04500 | -1.546 to 1.636 | No | ns | >0.9999 |
| SCC-25 vs. VU-147T3 | -0.07000 | -1.661 to 1.521 | No | ns | >0.9999 |
| SCC-25 vs. 2A3 | 0.01000 | -1.581 to 1.601 | No | ns | >0.9999 |
| CAL-27 vs. SCC-9 | 0.4392 | -1.339 to 2.218 | No | ns | 0.9796 |
| CAL-27 vs. FaDu | 0.4467 | -1.144 to 2.037 | No | ns | 0.9644 |
| CAL-27 vs. VU-147T3 | 0.3317 | -1.259 to 1.922 | No | ns | 0.9905 |
| CAL-27 vs. 2A3 | 0.4117 | -1.179 to 2.002 | No | ns | 0.9750 |
| SCC-9 vs. FaDu | 0.007500 | -1.771 to 1.786 | No | ns | >0.9999 |
| SCC-9 vs. VU-147T3 | -0.1075 | -1.886 to 1.671 | No | ns | >0.9999 |
| SCC-9 vs. 2A3 | -0.02750 | -1.806 to 1.751 | No | ns | >0.9999 |
| FaDu vs. VU-147T3 | -0.1150 | -1.706 to 1.476 | No | ns | >0.9999 |
| FaDu vs. 2A3 | -0.03500 | -1.626 to 1.556 | No | ns | >0.9999 |
| VU-147T3 vs. 2A3 | 0.08000 | -1.511 to 1.671 | No | ns | >0.9999 |
|  |  |  |  |  |  |

**Figure 4B**

| Paucimannose |  |  |  |  |  |
| --- | --- | --- | --- | --- | --- |
| SCC-25 vs. CAL-27 | 0.4500 | -1.276 to 2.176 | No | ns | 0.9716 |
| SCC-25 vs. SCC-9 | 0.2633 | -1.667 to 2.193 | No | ns | 0.9986 |
| SCC-25 vs. FaDu | 3.018 | 1.292 to 4.745 | Yes | **** | <0.0001 |
| SCC-25 vs. VU-147T3 | -0.5933 | -2.320 to 1.133 | No | ns | 0.9113 |
| SCC-25 vs. 2A3 | 0.5250 | -1.201 to 2.251 | No | ns | 0.9455 |
| CAL-27 vs. SCC-9 | -0.1867 | -2.117 to 1.743 | No | ns | 0.9997 |
| CAL-27 vs. FaDu | 2.568 | 0.8421 to 4.295 | Yes | *** | 0.0007 |
| CAL-27 vs. VU-147T3 | -1.043 | -2.770 to 0.6829 | No | ns | 0.4845 |
| CAL-27 vs. 2A3 | 0.07500 | -1.651 to 1.801 | No | ns | >0.9999 |
| SCC-9 vs. FaDu | 2.755 | 0.8251 to 4.685 | Yes | ** | 0.0012 |
| SCC-9 vs. VU-147T3 | -0.8567 | -2.787 to 1.073 | No | ns | 0.7785 |
| SCC-9 vs. 2A3 | 0.2617 | -1.668 to 2.192 | No | ns | 0.9986 |
| FaDu vs. VU-147T3 | -3.612 | -5.338 to -1.885 | Yes | **** | <0.0001 |
| FaDu vs. 2A3 | -2.493 | -4.220 to -0.7671 | Yes | ** | 0.0011 |
| VU-147T3 vs. 2A3 | 1.118 | -0.6079 to 2.845 | No | ns | 0.4060 |
|  |  |  |  |  |  |
| Paucimannose Fuc |  |  |  |  |  |
| SCC-25 vs. CAL-27 | 0.3717 | -1.355 to 2.098 | No | ns | 0.9878 |
| SCC-25 vs. SCC-9 | -0.2250 | -2.155 to 1.705 | No | ns | 0.9993 |
| SCC-25 vs. FaDu | 2.280 | 0.5538 to 4.006 | Yes | ** | 0.0034 |
| SCC-25 vs. VU-147T3 | -0.3600 | -2.086 to 1.366 | No | ns | 0.9895 |
| SCC-25 vs. 2A3 | 0.2233 | -1.503 to 1.950 | No | ns | 0.9989 |
| CAL-27 vs. SCC-9 | -0.5967 | -2.527 to 1.333 | No | ns | 0.9418 |
| CAL-27 vs. FaDu | 1.908 | 0.1821 to 3.635 | Yes | * | 0.0221 |
| CAL-27 vs. VU-147T3 | -0.7317 | -2.458 to 0.9945 | No | ns | 0.8098 |
| CAL-27 vs. 2A3 | -0.1483 | -1.875 to 1.578 | No | ns | 0.9998 |
| SCC-9 vs. FaDu | 2.505 | 0.5751 to 4.435 | Yes | ** | 0.0042 |
| SCC-9 vs. VU-147T3 | -0.1350 | -2.065 to 1.795 | No | ns | >0.9999 |
| SCC-9 vs. 2A3 | 0.4483 | -1.482 to 2.378 | No | ns | 0.9829 |
| FaDu vs. VU-147T3 | -2.640 | -4.366 to -0.9138 | Yes | *** | 0.0005 |
| FaDu vs. 2A3 | -2.057 | -3.783 to -0.3305 | Yes | * | 0.0108 |
| VU-147T3 vs. 2A3 | 0.5833 | -1.143 to 2.310 | No | ns | 0.9170 |

**Figure 4C**

| SCC-25 vs. CAL-27 | -0.6333 | -14.30 to 13.03 | No | ns | >0.9999 |
| --- | --- | --- | --- | --- | --- |
| SCC-25 vs. SCC-9 | -3.767 | -21.42 to 13.89 | No | ns | 0.8128 |
| SCC-25 vs. FaDu | 8.480 | 6.113 to 10.85 | Yes | *** | 0.0002 |
| SCC-25 vs. VU-147T3 | 21.93 | 13.26 to 30.59 | Yes | *** | 0.0008 |
| SCC-25 vs. 2A3 | 4.227 | -1.424 to 9.877 | No | ns | 0.1380 |
| CAL-27 vs. SCC-9 | -3.133 | -16.16 to 9.890 | No | ns | 0.7475 |
| CAL-27 vs. FaDu | 9.113 | -6.550 to 24.78 | No | ns | 0.2806 |
| CAL-27 vs. VU-147T3 | 22.56 | 17.14 to 27.98 | Yes | **** | <0.0001 |
| CAL-27 vs. 2A3 | 4.860 | -3.475 to 13.20 | No | ns | 0.2791 |
| SCC-9 vs. FaDu | 12.25 | -7.540 to 32.03 | No | ns | 0.1675 |
| SCC-9 vs. VU-147T3 | 25.69 | 16.59 to 34.80 | Yes | ** | 0.0025 |
| SCC-9 vs. 2A3 | 7.993 | -5.905 to 21.89 | No | ns | 0.1976 |
| FaDu vs. VU-147T3 | 13.45 | 2.686 to 24.21 | Yes | * | 0.0205 |
| FaDu vs. 2A3 | -4.253 | -12.13 to 3.622 | No | ns | 0.3346 |
| VU-147T3 vs. 2A3 | -17.70 | -21.30 to -14.10 | Yes | **** | <0.0001 |
|  |  |  |  |  |  |

**Figure 5A**

**Phosphate oligomannose**

| SCC-25 vs. CAL-27 | -2.318 | -2.929 to -1.708 | Yes | *** | 0.0001 |
| --- | --- | --- | --- | --- | --- |
| SCC-25 vs. SCC-9 | -4.872 | -7.807 to -1.936 | Yes | * | 0.0121 |
| SCC-25 vs. FaDu | -1.463 | -2.298 to -0.6290 | Yes | ** | 0.0046 |
| SCC-25 vs. VU-147T3 | -4.518 | -5.565 to -3.471 | Yes | **** | <0.0001 |
| SCC-25 vs. 2A3 | -3.262 | -5.893 to -0.6307 | Yes | * | 0.0212 |
| CAL-27 vs. SCC-9 | -2.553 | -4.796 to -0.3108 | Yes | * | 0.0351 |
| CAL-27 vs. FaDu | 0.8550 | -0.3939 to 2.104 | No | ns | 0.1808 |
| CAL-27 vs. VU-147T3 | -2.200 | -3.159 to -1.241 | Yes | ** | 0.0013 |
| CAL-27 vs. 2A3 | -0.9433 | -3.142 to 1.255 | No | ns | 0.5211 |
| SCC-9 vs. FaDu | 3.408 | -0.6345 to 7.451 | No | ns | 0.0785 |
| SCC-9 vs. VU-147T3 | 0.3533 | -1.380 to 2.087 | No | ns | 0.8345 |
| SCC-9 vs. 2A3 | 1.610 | -3.329 to 6.549 | No | ns | 0.5446 |
| FaDu vs. VU-147T3 | -3.055 | -4.877 to -1.233 | Yes | ** | 0.0057 |
| FaDu vs. 2A3 | -1.798 | -4.695 to 1.099 | No | ns | 0.2376 |
| VU-147T3 vs. 2A3 | 1.257 | -1.400 to 3.913 | No | ns | 0.4398 |
|  |  |  |  |  |  |

**Figure 5B**

| Man5 |  |  |  |  |  |
| --- | --- | --- | --- | --- | --- |
| SCC-25 vs. CAL-27 | -0.3217 | -1.873 to 1.230 | No | ns | 0.9910 |
| SCC-25 vs. SCC-9 | -0.3467 | -2.081 to 1.388 | No | ns | 0.9924 |
| SCC-25 vs. FaDu | -0.06500 | -1.617 to 1.487 | No | ns | >0.9999 |
| SCC-25 vs. VU-147T3 | -0.1917 | -1.743 to 1.360 | No | ns | 0.9992 |
| SCC-25 vs. 2A3 | -0.2117 | -1.763 to 1.340 | No | ns | 0.9987 |
| CAL-27 vs. SCC-9 | -0.02500 | -1.760 to 1.710 | No | ns | >0.9999 |
| CAL-27 vs. FaDu | 0.2567 | -1.295 to 1.808 | No | ns | 0.9969 |
| CAL-27 vs. VU-147T3 | 0.1300 | -1.422 to 1.682 | No | ns | 0.9999 |
| CAL-27 vs. 2A3 | 0.1100 | -1.442 to 1.662 | No | ns | >0.9999 |
| SCC-9 vs. FaDu | 0.2817 | -1.453 to 2.016 | No | ns | 0.9971 |
| SCC-9 vs. VU-147T3 | 0.1550 | -1.580 to 1.890 | No | ns | 0.9998 |
| SCC-9 vs. 2A3 | 0.1350 | -1.600 to 1.870 | No | ns | >0.9999 |
| FaDu vs. VU-147T3 | -0.1267 | -1.678 to 1.425 | No | ns | 0.9999 |
| FaDu vs. 2A3 | -0.1467 | -1.698 to 1.405 | No | ns | 0.9998 |
| VU-147T3 vs. 2A3 | -0.02000 | -1.572 to 1.532 | No | ns | >0.9999 |
|  |  |  |  |  |  |
| Man6 |  |  |  |  |  |
| SCC-25 vs. CAL-27 | -3.212 | -4.763 to -1.660 | Yes | **** | <0.0001 |
| SCC-25 vs. SCC-9 | -3.093 | -4.827 to -1.358 | Yes | **** | <0.0001 |
| SCC-25 vs. FaDu | -2.010 | -3.562 to -0.4583 | Yes | ** | 0.0035 |
| SCC-25 vs. VU-147T3 | -1.688 | -3.240 to -0.1367 | Yes | * | 0.0244 |
| SCC-25 vs. 2A3 | -3.912 | -5.463 to -2.360 | Yes | **** | <0.0001 |
| CAL-27 vs. SCC-9 | 0.1192 | -1.616 to 1.854 | No | ns | >0.9999 |
| CAL-27 vs. FaDu | 1.202 | -0.3500 to 2.753 | No | ns | 0.2273 |
| CAL-27 vs. VU-147T3 | 1.523 | -0.02832 to 3.075 | No | ns | 0.0575 |
| CAL-27 vs. 2A3 | -0.7000 | -2.252 to 0.8517 | No | ns | 0.7826 |
| SCC-9 vs. FaDu | 1.083 | -0.6523 to 2.817 | No | ns | 0.4671 |
| SCC-9 vs. VU-147T3 | 1.404 | -0.3306 to 3.139 | No | ns | 0.1857 |
| SCC-9 vs. 2A3 | -0.8192 | -2.554 to 0.9156 | No | ns | 0.7481 |
| FaDu vs. VU-147T3 | 0.3217 | -1.230 to 1.873 | No | ns | 0.9910 |
| FaDu vs. 2A3 | -1.902 | -3.453 to -0.3500 | Yes | ** | 0.0070 |
| VU-147T3 vs. 2A3 | -2.223 | -3.775 to -0.6717 | Yes | *** | 0.0008 |
|  |  |  |  |  |  |
| Man7 |  |  |  |  |  |
| SCC-25 vs. CAL-27 | -0.4867 | -2.038 to 1.065 | No | ns | 0.9443 |
| SCC-25 vs. SCC-9 | 1.663 | -0.07147 to 3.398 | No | ns | 0.0683 |
| SCC-25 vs. FaDu | -1.188 | -2.740 to 0.3633 | No | ns | 0.2384 |
| SCC-25 vs. VU-147T3 | -0.4383 | -1.990 to 1.113 | No | ns | 0.9641 |
| SCC-25 vs. 2A3 | -4.580 | -6.132 to -3.028 | Yes | **** | <0.0001 |
| CAL-27 vs. SCC-9 | 2.150 | 0.4152 to 3.885 | Yes | ** | 0.0062 |
| CAL-27 vs. FaDu | -0.7017 | -2.253 to 0.8500 | No | ns | 0.7809 |
| CAL-27 vs. VU-147T3 | 0.04833 | -1.503 to 1.600 | No | ns | >0.9999 |
| CAL-27 vs. 2A3 | -4.093 | -5.645 to -2.542 | Yes | **** | <0.0001 |
| SCC-9 vs. FaDu | -2.852 | -4.586 to -1.117 | Yes | **** | <0.0001 |
| SCC-9 vs. VU-147T3 | -2.102 | -3.836 to -0.3669 | Yes | ** | 0.0080 |
| SCC-9 vs. 2A3 | -6.243 | -7.978 to -4.509 | Yes | **** | <0.0001 |
| FaDu vs. VU-147T3 | 0.7500 | -0.8017 to 2.302 | No | ns | 0.7290 |
| FaDu vs. 2A3 | -3.392 | -4.943 to -1.840 | Yes | **** | <0.0001 |
| VU-147T3 vs. 2A3 | -4.142 | -5.693 to -2.590 | Yes | **** | <0.0001 |
|  |  |  |  |  |  |
| Man8 |  |  |  |  |  |
| SCC-25 vs. CAL-27 | 8.000 | 6.448 to 9.552 | Yes | **** | <0.0001 |
| SCC-25 vs. SCC-9 | 9.095 | 7.360 to 10.83 | Yes | **** | <0.0001 |
| SCC-25 vs. FaDu | 4.940 | 3.388 to 6.492 | Yes | **** | <0.0001 |
| SCC-25 vs. VU-147T3 | 8.602 | 7.050 to 10.15 | Yes | **** | <0.0001 |
| SCC-25 vs. 2A3 | 3.970 | 2.418 to 5.522 | Yes | **** | <0.0001 |
| CAL-27 vs. SCC-9 | 1.095 | -0.6398 to 2.830 | No | ns | 0.4539 |
| CAL-27 vs. FaDu | -3.060 | -4.612 to -1.508 | Yes | **** | <0.0001 |
| CAL-27 vs. VU-147T3 | 0.6017 | -0.9500 to 2.153 | No | ns | 0.8722 |
| CAL-27 vs. 2A3 | -4.030 | -5.582 to -2.478 | Yes | **** | <0.0001 |
| SCC-9 vs. FaDu | -4.155 | -5.890 to -2.420 | Yes | **** | <0.0001 |
| SCC-9 vs. VU-147T3 | -0.4933 | -2.228 to 1.241 | No | ns | 0.9631 |
| SCC-9 vs. 2A3 | -5.125 | -6.860 to -3.390 | Yes | **** | <0.0001 |
| FaDu vs. VU-147T3 | 3.662 | 2.110 to 5.213 | Yes | **** | <0.0001 |
| FaDu vs. 2A3 | -0.9700 | -2.522 to 0.5817 | No | ns | 0.4650 |
| VU-147T3 vs. 2A3 | -4.632 | -6.183 to -3.080 | Yes | **** | <0.0001 |
|  |  |  |  |  |  |
| Man9 |  |  |  |  |  |
| SCC-25 vs. CAL-27 | 0.9783 | -0.5733 to 2.530 | No | ns | 0.4551 |
| SCC-25 vs. SCC-9 | -2.298 | -4.032 to -0.5627 | Yes | ** | 0.0026 |
| SCC-25 vs. FaDu | 1.367 | -0.1850 to 2.918 | No | ns | 0.1183 |
| SCC-25 vs. VU-147T3 | 1.835 | 0.2833 to 3.387 | Yes | * | 0.0105 |
| SCC-25 vs. 2A3 | 0.4767 | -1.075 to 2.028 | No | ns | 0.9489 |
| CAL-27 vs. SCC-9 | -3.276 | -5.011 to -1.541 | Yes | **** | <0.0001 |
| CAL-27 vs. FaDu | 0.3883 | -1.163 to 1.940 | No | ns | 0.9788 |
| CAL-27 vs. VU-147T3 | 0.8567 | -0.6950 to 2.408 | No | ns | 0.6030 |
| CAL-27 vs. 2A3 | -0.5017 | -2.053 to 1.050 | No | ns | 0.9370 |
| SCC-9 vs. FaDu | 3.664 | 1.929 to 5.399 | Yes | **** | <0.0001 |
| SCC-9 vs. VU-147T3 | 4.132 | 2.398 to 5.867 | Yes | **** | <0.0001 |
| SCC-9 vs. 2A3 | 2.774 | 1.039 to 4.509 | Yes | *** | 0.0001 |
| FaDu vs. VU-147T3 | 0.4683 | -1.083 to 2.020 | No | ns | 0.9525 |
| FaDu vs. 2A3 | -0.8900 | -2.442 to 0.6617 | No | ns | 0.5621 |
| VU-147T3 vs. 2A3 | -1.358 | -2.910 to 0.1933 | No | ns | 0.1226 |

**Figure 6A**

**Oligomannose Fucose**

| SCC-25 vs. CAL-27 | -0.1567 | -0.2067 to -0.1066 | Yes | *** | 0.0003 |
| --- | --- | --- | --- | --- | --- |
| SCC-25 vs. SCC-9 | -0.6433 | -1.089 to -0.1977 | Yes | * | 0.0180 |
| SCC-25 vs. FaDu | -0.09333 | -0.1563 to -0.03038 | Yes | ** | 0.0098 |
| SCC-25 vs. VU-147T3 | -0.3483 | -0.4531 to -0.2436 | Yes | *** | 0.0002 |
| SCC-25 vs. 2A3 | -0.2433 | -0.6640 to 0.1773 | No | ns | 0.2847 |
| CAL-27 vs. SCC-9 | -0.4867 | -0.9343 to -0.03902 | Yes | * | 0.0398 |
| CAL-27 vs. FaDu | 0.06333 | 0.0003801 to 0.1263 | Yes | * | 0.0489 |
| CAL-27 vs. VU-147T3 | -0.1917 | -0.2946 to -0.08868 | Yes | ** | 0.0035 |
| CAL-27 vs. 2A3 | -0.08667 | -0.4595 to 0.2861 | No | ns | 0.9029 |
| SCC-9 vs. FaDu | 0.5500 | 0.1682 to 0.9318 | Yes | * | 0.0181 |
| SCC-9 vs. VU-147T3 | 0.2950 | -0.09120 to 0.6812 | No | ns | 0.1009 |
| SCC-9 vs. 2A3 | 0.4000 | -0.4072 to 1.207 | No | ns | 0.2706 |
| FaDu vs. VU-147T3 | -0.2550 | -0.3190 to -0.1910 | Yes | **** | <0.0001 |
| FaDu vs. 2A3 | -0.1500 | -0.5590 to 0.2590 | No | ns | 0.6481 |
| VU-147T3 vs. 2A3 | 0.1050 | -0.3157 to 0.5257 | No | ns | 0.8770 |

**References**

1. Jensen PH, Karlsson NG, Kolarich D, Packer NH (2012) Structural analysis of N- and O-glycans released from glycoproteins. Nat Protoc 7 (7):1299-1310. doi:10.1038/nprot.2012.063

2. Oliveira T, Zhang M, Joo EJ, Abdel-Azim H, Chen CW, Yang L, Chou CH, Qin X, Chen J, Alagesan K, Almeida A, Jacob F, Packer NH, von Itzstein M, Heisterkamp N, Kolarich D (2021) Glycoproteome remodeling in MLL-rearranged B-cell precursor acute lymphoblastic leukemia. Theranostics 11 (19):9519-9537. doi:10.7150/thno.65398

3. Everest-Dass AV, Abrahams JL, Kolarich D, Packer NH, Campbell MP (2013) Structural feature ions for distinguishing N- and O-linked glycan isomers by LC-ESI-IT MS/MS. J Am Soc Mass Spectrom 24 (6):895-906. doi:10.1007/s13361-013-0610-4

4. Stanley P, Moremen KW, Lewis NE, Taniguchi N, Aebi M (2022) N-Glycans. In: th, Varki A, Cummings RD et al. (eds) Essentials of Glycobiology. Cold Spring Harbor (NY), pp 103-116. doi:10.1101/glycobiology.4e.9

5. Hinneburg H, Korać P, Schirmeister F, Gasparov S, Seeberger PH, Zoldoš V, Kolarich D (2017) Unlocking Cancer Glycomes from Histopathological Formalin-fixed and Paraffin-embedded (FFPE) Tissue Microdissections. Mol Cell Proteomics 16 (4):524-536. doi:10.1074/mcp.M116.062414

6. Ceroni A, Maass K, Geyer H, Geyer R, Dell A, Haslam SM (2008) GlycoWorkbench: a tool for the computer-assisted annotation of mass spectra of glycans. J Proteome Res 7 (4):1650-1659. doi:10.1021/pr7008252
